# Improved hydrolysis of piperacillin by OXA-48-like R214G variants, a selective advantage under piperacillin-tazobactam exposure

**DOI:** 10.1128/aac.01267-25

**Published:** 2026-04-29

**Authors:** Réva Nermont, Saoussen Oueslati, Magali Aumont-Nicaise, Pascal Retailleau, Bogdan I. Iorga, Thierry Naas

**Affiliations:** 1 Faculty of Medicine, Team 'Resist', UMR1184 'Immunology of Viral, Auto-Immune, Hematological and Bacterial Diseases (IMVA-HB),' INSERM, Université Paris-Saclay, CEA, LabEx LERMIT 26930, Le Kremlin-Bicêtre, France; 2 Bacteriology-Hygiene Department, Hôpital Bicêtre, AP-HP Paris-Saclay 41664, Le Kremlin-Bicêtre, France; 3 Université Paris-Saclay, CNRS, Plateforme Interactions des Macromolécules Institut de Biologie Intégrative de la Cellule (I2BC)-UMR9198 531573, Gif-sur-Yvette, France; 4 Université Paris-Saclay, CNRS, Institut de Chimie des Substances Naturelles (ICSN) 57474, Gif-sur-Yvette, France; 5 French National Reference Center for Antibiotic Resistance: Carbapenemase-Producing Enterobacterales, Hôpital Bicêtre, AP-HP Paris-Saclay 378965 , Le Kremlin-Bicêtre, France; Università degli Studi di Roma “La Sapienza”, Rome, Italy

**Keywords:** loss of function, carbapenemase, OXA-48

## Abstract

OXA-48-like carbapenemases have rapidly disseminated worldwide, becoming the most common carbapenemase in many countries, with more than 60 variants reported. Among them, OXA-244 (OXA-48-R214G) and OXA-484 (OXA-181-R214G) are increasingly reported, despite overall reduced hydrolytic activities for β-lactams, including temocillin and carbapenems. R214, located in the β5–β6 loop, through the interaction with D159, is crucial for carbapenem hydrolysis by structuring the active site. R214G variants of OXA-48-likes were analyzed by β-lactam susceptibility testing, steady-state kinetic analyses in the presence or absence of sodium hydrogen carbonate (NaHCO_3_), molecular modeling, X-ray crystallography, and protein stability assessments using differential scanning fluorimetry (DSF). The R214G substitution in OXA-48 and OXA-181 results in reduced minimal inhibitory concentrations (MICs) for all β-lactams, except for piperacillin and piperacillin/tazobactam combination, for which MICs are increased. OXA-244 and OXA-484 displayed a better affinity for piperacillin with lower *K_m_* than for parental enzymes and thus resulted in a higher catalytic efficiency for piperacillin hydrolysis. These results were supported by docking observations, highlighting enhanced affinity when glycine is located at position 214. Overall, DSF indicated that the R214G substitution, while destabilizing the active site, enhances the global stability of the protein, especially for OXA-244. The addition of NaHCO_3_ raised the activity and thermal stability of all enzymes, especially of OXA-48, which appeared more sensitive to the presence of NaHCO_3_. Although OXA-48-like R214G is considered a loss-of-function variant, our findings indicate it exhibits increased hydrolytic activity toward piperacillin, which may result in an advantage under piperacillin-tazobactam exposure and thus could contribute to the selection of these globally expanding variants.

## INTRODUCTION

OXA-48 carbapenemases have rapidly disseminated worldwide to become one of the most common carbapenemases with more than 60 variants reported, with, in some cases, significant differences in their hydrolysis profiles ([1]; http://bldb.eu/BLDB.php?class=D#OXA, accessed on 18 August 2025). These variants can be classified into four groups according to their hydrolysis profile. Most of them, including OXA-181 or OXA-162, have an enzymatic activity similar to OXA-48, i.e., hydrolysis of penicillins including temocillin, narrow-spectrum cephalosporins, and also carbapenems at a low rate, but spares expanded-spectrum cephalosporins (ESC) (2). The second group, represented by OXA-163, OXA-247, and OXA-405 (2, 3), has no carbapenemase activity but instead has an improved hydrolytic activity against ESC, similar to OXA-ESBLs (4). The third group has carbapenem and ESC hydrolytic activity, such as OXA-517 and OXA-438 (5). The fourth group, which possesses amino acid (AA) changes at position R214, such as OXA-48 R214G (OXA-244), OXA-181 R214G (OXA-484), and OXA-181 R214S (OXA-232), is the most intriguing variants, as they display overall reduced hydrolytic activities including carbapenems and temocillin, and yet they are increasingly isolated worldwide (6–8). Position R214 in the β5–β6 loop is crucial for carbapenem and temocillin hydrolysis activity, as it stabilizes these antibiotics in the active site and maintains the shape of the latter through interactions with D159, itself thought to be stabilized by S155 (6–10). OXA-48 D159N (OXA-933) also displays reduced enzymatic activity, likely because of destabilizing the salt bridge with R214, thus destabilizing the active site. It has only been described once in a *Klebsiella pneumoniae* isolate from France (10).

Possible explanations for their spread are likely multifactorial. Indeed, it has been suggested that, as temocillin and carbapenems are present in several screening media used for the detection of carbapenemase-producing enterobacterales (CPEs), reduced hydrolysis of these molecules can result in difficulties in their detection, which may lead to under-detection and silent spread (11–13). In addition, R214G/S variants have been found associated with high-risk clones, further supporting their efficient spread. This is the case for OXA-181 R214G (OXA-484), identified predominantly in two sequence types in *Escherichia coli*: ST410 and ST1722; OXA-48 R214G (OXA-244), frequently found in *E. coli* ST38; and OXA-181 R214S (OXA-232), frequently found in two *K. pneumoniae* high-risk clones, ST-231 and ST-2096 (14–16). It is, however, unclear what could have triggered the emergence of these variants with an altered substrate profile (mostly loss of function), as bacteria producing these variants have overall reduced minimal inhibitory concentrations (MICs) to all tested antibiotics, as compared to OXA-48 producers (6).

Here, we have investigated possible β-lactam substrates that might have been at the origin of the selection of these variants. We have conducted susceptibility testing, steady-state kinetic measurements, and molecular modeling using various β-lactam substrates, including piperacillin-tazobactam, a widely used antibiotic in clinics that has been incriminated in the emergence of broad-spectrum β-lactam resistance in *E. coli* (17–19).

## RESULTS

### Susceptibility testing

Overall, increased inhibition diameters were observed as compared to bacteria producing OXA-48 or OXA-181, except for piperacillin and piperacillin/tazobactam, which revealed reduced or unchanged inhibition diameters for R214G variants of OXA-48 and OXA-181, respectively (Table 1). With OXA-48 D159N (OXA-933) and OXA-181 R214S (OXA-232), which are also variants with altered substrate profiles, diameters for all the β-lactams tested are increased (Table 1).

**TABLE 1:** Routine disc diffusion antibiograms

Antibiotics	Diameters in mm (EUCAST interpretation)^*a*^						
	*E. coli* TOP10 pTOPO-						*E. coli* TOP10
	OXA-48	OXA-48 R214G (OXA-244)	OXA-48 D159N (OXA-933)	OXA-181	OXA-181 R214G (OXA-484)	OXA-181 R214S (OXA-232)	
Amoxicillin	6 (R)	6 (R)	17 (S)	6 (R)	6 (R)	6 (R)	24 (S)
Amoxicillin-clavulanic acid	6 (R)	6 (R)	19 (S)	6 (R)	6 (R)	6 (R)	26 (S)
Ticarcillin	6	6	14	6	6	6	30
Piperacillin	17 (R)	**12 (R**)^*b*^	27 (S)	8 (R)	**8 (R**)	15 (R)	26 (S)
Piperacillin-tazobactam	19 (R)	**14 (R**)	29 (S)	14 (R)	**14 (R**)	21 (R)	25 (S)
Temocillin	6 (R)	17 (I)	17 (I)	6 (R)	18 (I)	18 (I)	24 (I)
Cephalothin	16	19	19	17	18	19	25
Cephalexin	21 (S)	25 (S)	25 (S)	24 (S)	24 (S)	24 (S)	25 (S)
Cefoxitin	27 (S)	27 (S)	27 (S)	27 (S)	27 (S)	27 (S)	27 (S)
Ceftazidime	28 (S)	31 (S)	32 (S)	28 (S)	30 (S)	31 (S)	30 (S)
Ceftazidime/avibactam	27 (S)	30 (S)	30 (S)	30 (S)	31 (S)	30 (S)	30 (S)
Cefotaxime	30 (S)	33 (S)	36 (S)	30 (S)	34 (S)	34 (S)	35 (S)
Cefepime	35 (S)	35 (S)	35 (S)	35 (S)	35 (S)	35 (S)	35 (S)
Aztreonam	35 (S)	35 (S)	35 (S)	35 (S)	35 (S)	35 (S)	35 (S)
Imipenem	29 (S)	35 (S)	35 (S)	29 (S)	35 (S)	35 (S)	35 (S)
Meropenem	30 (S)	35 (S)	35 (S)	30 (S)	35 (S)	35 (S)	35 (S)
Ertapenem	30 (S)	35 (S)	35 (S)	24 (S)	30 (S)	30 (S)	37 (S)

^*a*^ 2025 EUCAST guidelines were used to interpret the Kirby-Bauer antibiogram (http://www.eucast.org). For Ticarcillin and cephalothin, no breakpoints are available.

^*b*^ In bold are diameters that are reduced as compared to bacteria producing parental enzymes.

MIC values for *E. coli* TOP10 expressing OXA-48 variants revealed that R214G substitutions in OXA-48 and OXA-181 conferred higher MIC for piperacillin, 128 µg/mL as compared to 32 µg/mL and 256 µg/mL as compared to 128 µg/mL, respectively (Table 2). With the piperacillin-tazobactam combination, the R214G substitution conferred higher MICs, with an eight- and twofold increase as compared to their parental enzymes, OXA-48 and OXA-181, respectively. However, the D159N substitution in OXA-48 and the R214S substitution in OXA-181 resulted in reduced MICs for piperacillin and piperacillin-tazobactam as compared to OXA-48 and OXA-181, respectively. All the tested variants have a drastic reduction in MICs for temocillin and carbapenems, as previously shown (6–8, 20).

**TABLE 2:** Carba NP assay, NG-Test Carba5 results, and MICs of β-lactams for *E. coli* TOP10 expressing OXA-48, OXA-48 R214G (OXA-244), OXA-48 D159N (OXA-933), OXA-181, OXA-181 R214G (OXA-484), and OXA-181 R214S (OXA-232)^*c*^

Antibiotics	*E. coli* TOP10-pTOPO-						*E. coli* TOP10	*E. coli* HB4-pTOPO-						*E. coli* HB4
	OXA- 48	OXA- 48 R214G (OXA-244)	OXA- 48 D159N (OXA-933)	OXA- 181	OXA- 181 R214G (OXA-484)	OXA- 181 R214S (OXA-232)	–	OXA- 48	OXA- 48 R214G (OXA-244)	OXA- 48 D159N (OXA-933)	OXA- 181	OXA- 181 R214G (OXA-484)	OXA- 181 R214S (OXA-232)	–
Amoxicillin	>256	>256	32	>256	>256	>256	3	>256	>256	>256	>256	>256	>256	12
Amoxicillin-clavulanic acid	>256	>256	16	>256	>256	>256	2	>256	>256	>256	>256	>256	>256	12
Piperacillin^*a*^	32	**128^*b*^**	8	128	**256**	16	4	**128**	**512**	8	**256**	**1024**	128	8
Piperacillin-tazobactam^*a*^	2	**16**	2	32	**64**	4	2	**128**	**512**	8	**256**	**512**	128	4
Temocillin	>1024	16	12	>1024	12	16	4	>1024	>1024	>1024	>1024	>1024	>1024	24
Ceftazidime	0.25	0.25	0.25	0.25	0.38	0.25	0.25	1	0.75	0.75	1	2	0.75	1
Cefotaxime	0.19	0.064	0.064	0.25	0.094	0.064	0.064	2	0.75	0.5	2	1	0.75	0.5
Cefepime	0.064	0.064	0.047	0.125	0.047	0.032	0.032	2	1	1	2	1.5	1.5	1
Imipenem	0.5	0.25	0.25	0.75	0.25	0.25	0.19	>32	>32	0.19	>32	>32	>32	0.19
Meropenem	0.047	0.032	0.023	0.125	0.023	0.023	0.023	>32	>32	0.5	>32	>32	>32	0.25
Ertapenem	0.125	0.023	0.004	0.25	0.064	0.012	0.003	>32	>32	0.25	>32	>32	>32	0.25
Carba NP test	+	−	−	+	−	−	−	+	−	−	+	±	±	−
NG-Test Carba 5	OXA	OXA	OXA	OXA	OXA	OXA	–	OXA	OXA	OXA	OXA	OXA	OXA	–

^*a*^ Broth microdilution MIC results.

^*b*^ In bold are MICs that are increased as compared to bacteria producing parental enzymes.

^*c*^ +, positive test result; -, negative test result; +/-, inconclusive test result.

Susceptibility tests performed in *E. coli* HB4, a strain lacking OmpF and OmpC porins, revealed higher MICs for all the β-lactam tested than those obtained for *E. coli* TOP10 irrespective of the OXA-48-like variants, except for OXA-48-D159N (OXA-933), where only the MICs for amoxicillin, amoxicillin/clavulanate acid, and temocillin were significantly increased, unlike carbapenems. The MICs for piperacillin and piperacillin/tazobactam were higher as compared to those obtained in *E. coli* TOP10, with, however, a one- to twofold dilution persisting between the R214G variants and their respective parental enzymes. Regarding OXA-181 R214S (OXA-232), the MICs for piperacillin and piperacillin/tazobactam are similar to those of OXA-48.

### Steady-state kinetics

The catalytic efficiency toward benzylpenicillin increases overall in the presence of sodium hydrogen carbonate (NaHCO_3_), driven by higher *k_cat_* values for OXA-48, OXA-48-R214G (OXA-244), and OXA-181-R214S (OXA-232; 1.8- to 2.3-fold), whereas the catalytic efficiency of OXA-181 and OXA-181-R214G (OXA-484) does not appear to be affected by the presence of NaHCO_3_ (Table 3). In contrast, OXA-48-D159N (OXA-933) exhibits a lower *K_m_* in the presence of NaHCO_3_ (2.6-fold).

**TABLE 3:** Steady-state kinetic parameters for hydrolysis of β-lactam substrates by OXA-48-like β-lactamases

		*K_m_* (µM)^*a*^						*k*_*cat*_(*s*^-1^)^*a*^						(*k*_*cat*_*/K*_*m*_(mM^-1^*s*^-1^)^*a*^					
Substrate	Buffer	OXA-48	OXA-48 R214G (OXA-244)	OXA-48 D159N (OXA-933)	OXA-181	OXA-181 R214G (OXA-484)	OXA-181 R214S (OXA-232)	OXA-48	OXA-48 R214G (OXA-244)	OXA-48 D159N (OXA-933)	OXA-181	OXA-181 R214G (OXA-484)	OXA-181 R214S (OXA-232)	OXA-48	OXA-48 R214G (OXA-244)	OXA-48 D159N (OXA-933)	OXA-181	OXA-181 R214G (OXA-484)	OXA-181 R214S (OXA-232)
Benzylpenicillin	Sodium phosphate buffer	111 (±7)	138 (±21)	284 (±5)	104 (±21)	108 (±15)	116 (±2)	109 (±13)	87 (±8)	278 (±50)	183 (±41)	180 (±36)	195 (±31)	980 (±55)	639 (±44)	981 (±181)	1759 (±44)	1660 (±128)	1687 (±295)
	Sodium phosphate buffer + NaHCO_3_	177 (±25)	206 (±53)	108 (±6)	108 (±10)	114 (±1)	143 (±25)	193 (±18)	188 (±31)	186 (±28)	242 (±36)	214 (±10)	454 (±67)	1100 (±80)	929 (±111)	1714 (±193)	2236 (±140)	1885 (±99)	3192 (±167)
Ratio sodium phosphate + NaHCO_3_/sodium phosphate		1.6	2.4	0.4	1	1	1.2	1.8	2.2	0.7	1.3	1.2	2.3	1.1	1.5	1.7	1.3	1.1	1.9
Piperacillin	Sodium phosphate buffer	373 (±69)	131 (±24)	223 (±62)	214 (±17)	123 (±17)	214 (±20)	33 (±3)	75 (±13)	69 (±12)	48 (±7)	74 (±12)	168 (±3)	93 (±20)	576 (±33)	316 (±40)	224 (±16)	605 (±36)	786 (±57)
	Sodium phosphate buffer + NaHCO_3_	348 (±14)	160 (±0.9)	396 (±30)	337 (±75)	101 (±38)	322 (±90)	161 (±10)	122 (±6)	206 (±41)	284 (±53)	232 (±70)	431 (±58)	463 (±13)	765 (±37)	510 (±116)	849 (±42)	2345 (±235)	1382 (±228)
Ratio sodium phosphate + NaHCO_3_/sodium phosphate		0.9	1.2	1.8	1.6	0.8	1.5	4.8	1.6	3	5.9	3.1	2.6	4.9	1.3	1.6	3.8	3.9	1.8
Piperacillin fold change as compared to parental enzyme	Sodium phosphate buffer		0.35	0.60		0.57	1.0		2.27	2.09		1.54	3.5		6.2	3.4		2.7	3.5
	Sodium phosphate buffer + NaHCO_3_		0.45	1.1		0.3	0.96		0.8	1.3		0.8	1.5		1.7	1.1		2.8	1.6

^*a*^ Data are the means of three independent experiments.

The *K_m_* for piperacillin of OXA-48 R214G (OXA-244) and OXA-181 R214G (OXA-484) was 2.2- and 3.3-fold lower than that of OXA-48 and OXA-181, respectively, suggesting a better affinity for piperacillin, which resulted in a 1.7- and 2.8-fold higher catalytic efficiency for piperacillin as compared to the values for OXA-48 and OXA-181, respectively. A similar trend in catalytic efficiency for the same enzymes was observed in the absence of NaHCO_3_. Interestingly, the D159N substitution in OXA-48 (OXA-933), which resulted in an almost susceptible phenotype when expressed in *E. coli*, had no impact on the catalytic efficiency for piperacillin, suggesting enzymatic stability problems *in vivo* for this variant. Interestingly, OXA-181 R214S (OXA-232) can hydrolyze piperacillin more efficiently than OXA-181, and yet when expressed in *E. coli*, it results in lower MICs for piperacillin, suggesting also stability issues *in vivo*. Notably, the hydrolysis of piperacillin is markedly increased in the presence of NaHCO_3_, as revealed by increased *k*_*cat*_ for OXA-48 and OXA-181 by 4.7- and 5.9-fold, respectively.

The 50% inhibitory concentration (IC50) values revealed that the most effective inhibitors tested against all variants were avibactam and tazobactam, unlike clavulanic acid, vaborbactam, relebactam, or enmetazobactam (Table 4). IC50 values for the R214G/S variants were similar to those of the parental enzymes, confirming that they are comparably inhibited by the different inhibitors, especially tazobactam.

**TABLE 4:** IC50 values of β-lactamase inhibitors against OXA-48-like β-lactamases

Inhibitors	IC50 (µM)^*a*^					
	OXA-48	OXA-48 R214G (OXA-244)	OXA-48 D159N (OXA-933)	OXA-181	OXA-181 R214G (OXA-484)	OXA-181 R214S (OXA-232)
Clavulanic acid	21	45	20	29	22	20
Tazobactam	5	6	9	6	4	5
Enmetazobactam	34	50	26	49	65	19
Avibactam	3	10	7	6	8	5
Relebactam	63	58	59	41	59	42
Vaborbactam	10	22	25	48	55	17

^*a*^ IC50 was determined in 100 mM sodium phosphate buffer (pH 7) supplemented by 25 mM sodium hydrogen carbonate (NaHCO_3_) and 100 µM benzylpenicillin as a reporter substrate.

### Molecular modeling and X-ray crystallography

**Fig 1:**
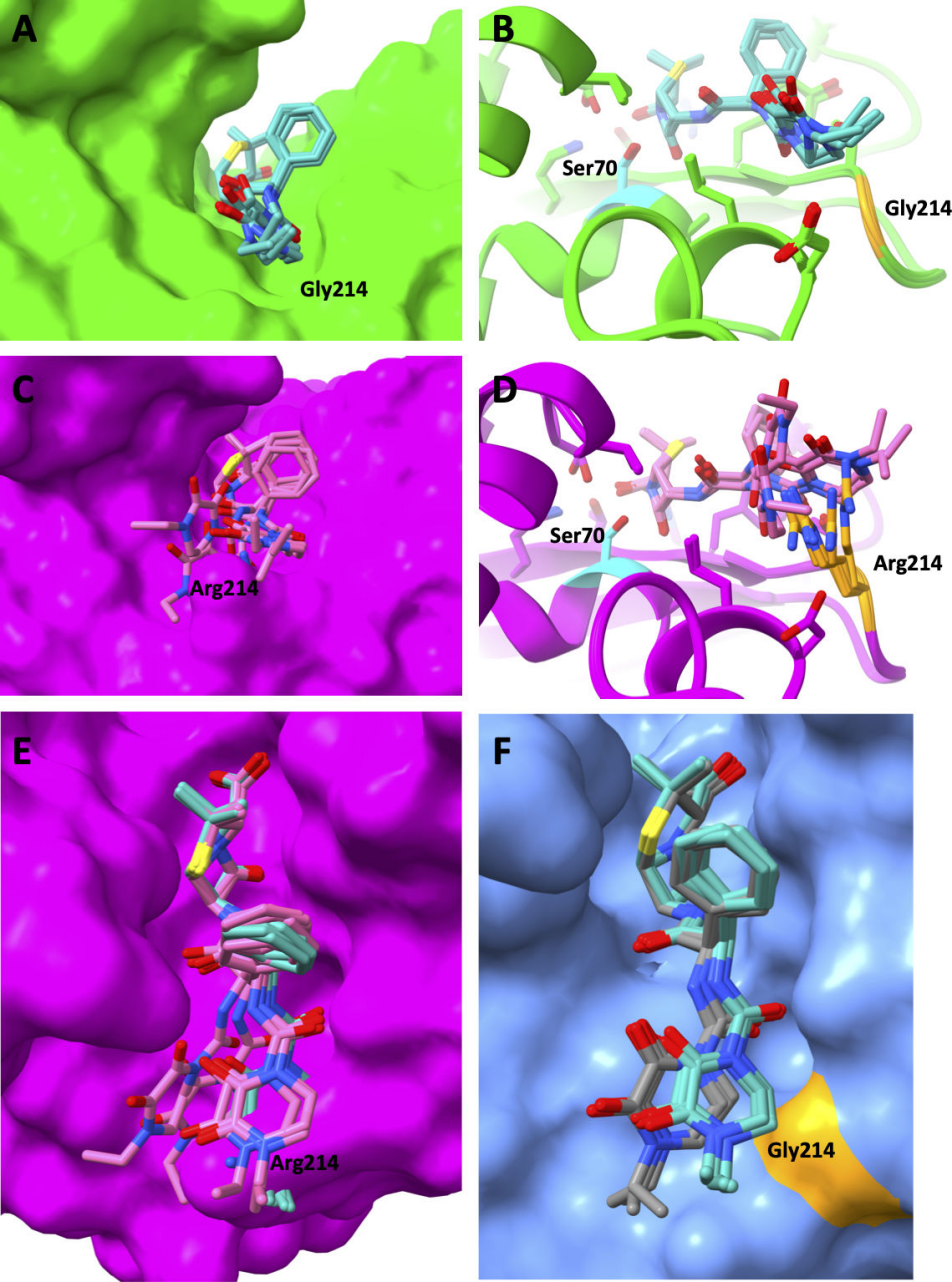
Complexes of OXA-48, OXA-244 (OXA-48 R214G), and OXA-1167 (OXA-48-R214S) with piperacillin predicted by AI co-folding. (**A and B**) OXA-244 (green) interacting with piperacillin (cyan): (**A**) surface display and (**B**) stick representations. (**C and D**) OXA-48 (magenta) interacting with piperacillin (pink): (**C**) surface display and (**D**) stick representations showing clashes with R214. (**E**) Piperacillin (pink) in the active site of OXA-48 displaying clashes with R214, piperacillin (cyan) as positioned in OXA-244, and surface of OXA-48 (magenta). (**F**) Piperacillin (cyan) in the active site of OXA-244, piperacillin (gray) as positioned in OXA-1167 with a different conformation to avoid clashes with S214, and surface of OXA-244 (blue) with Gly214 colored in orange. In panels **B** and **D**, Ser70 (cyan) is positioned in front of the β-lactam ring, compatible with a nucleophilic attack.

An *in silico* study was performed to identify the structural determinants that could explain the experimentally determined differences for piperacillin hydrolysis between the R214G variants and their parental enzymes. In all predictions, the β-lactam ring is situated as expected in front of the S70, in a position compatible with the nucleophilic attack (Fig. 1). As previously shown, the salt bridge present in OXA-48 between D159 and R214 is lost in R214G derivatives, resulting in reduced overall activity for most substrates (6–8, 20). However, piperacillin fits better into the active site of the R214G variants, taking advantage of the space originally occupied by R214 and thus allowing better hydrolysis. In OXA-48, the positioning of piperacillin is suboptimal, with clashes observed with the side chain of R214. Here, we show, using artificial intelligence (AI) co-folding calculations, that, unlike most substrates, piperacillin binding is favored in the absence of R214 (Fig. 1).

**Fig 2:**
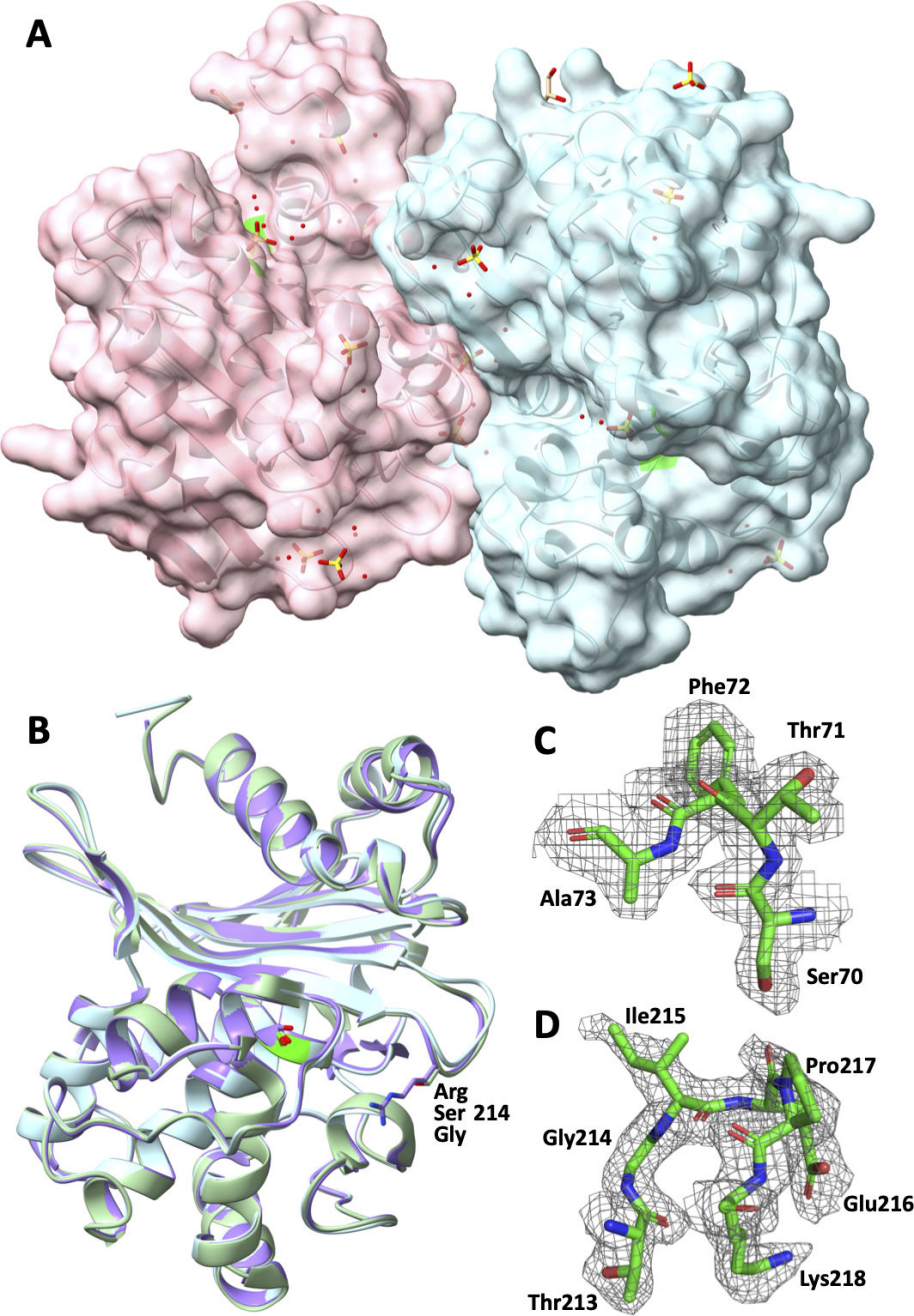
Crystal structure of OXA-244 K73A. (**A**) Surface representation of the crystallographic dimer. Ser70 is colored in green. (**B**) Superposed structures of OXA-244 K73A (cyan, PDB 9T06, this study), OXA-48 (purple, PDB 7LXG), and OXA-232 (green, PDB 5HFO) showing a conformational change of the β5-β6 loop in OXA-244. (**C and D**) 2Fo-Fc electron density around residues 70–73 (STFK, **C**) and 213–218 (TGIEPK, **D**) in the structure OXA-244 K73A (PDB 9T06, this study).

To better understand the effect of the R214G mutation in the OXA-48-like family, we determined the crystal structure of the OXA-48 R214G K73A (OXA-244 K73A) mutant at a 1.84 Å resolution (Fig. 2C and D). The structure is a crystallographic dimer (Fig. 2A), with a sulfate ion at the dimeric interface stabilized by ionic interactions with Arg206 from the two monomers. The overall fold is very similar to the other structures available from this family (1) (http://www.bldb.eu/S-BLDB.php), and the K73A does not induce significant conformational changes in the active site. A comparison with the structures of OXA-48 and OXA-48 R214S (OXA-1167) shows a conformational change of the β5-β6 loop in OXA-244 compared with the other two structures (Fig. 2B).

### Protein stability

The influence of the AA substitution on the thermal denaturation of OXA-48- and OXA-181-derivatives was evaluated using nano DSF using a Tycho NT.6 (NanoTemper Technologies, Munich, Germany) by measuring the intrinsic fluorescence of tryptophan as recommended by the manufacturer. Even though the R214G change loosens the active site of OXA-48, it increases the overall thermal stability of the enzyme by 4.8°C (Table 5). In contrast, the D159N change, which also destabilizes the active site, has a 2.8°C decrease in melting point. In the presence of NaHCO_3_, the stability of all the proteins was increased, especially for OXA-48 (increase of 11.2°C). With OXA-181 derivatives, the R214G change slightly reduces the thermal stability (2.8°C) (Table 5). The addition of NaHCO_3_ has only reduced the impact on the thermal stability of OXA-181 derivatives.

**TABLE 5:** Thermal denaturation of OXA-48- and OXA-181-derivatives as evaluated using differential scanning fluorimetry (DSF)

	Tm (°C)^*a*^					
Enzyme	OXA-48	OXA-48 R214G (OXA-244)	OXA-48 D159N (OXA-933)	OXA-181	OXA-181 R214G (OXA-484)	OXA-181 R214S (OXA-232)
Sodium phosphate buffer	60.6	65.4	57.7	61	58.2	59.5
Sodium phosphate buffer with NaHCO_3_^*b*^	71.8	68.8	69	62.4	60.7	62.1

^*a*^ First derivative of fluorescence ratio at 350 nm/330 nm of melting curves determined using DSF was used to calculate the melting temperature. All measurements were performed with 0.5 mg/mL proteins in 100 mM sodium phosphate buffer (pH 7.4) in the absence and presence of 25 mM sodium hydrogen carbonate (NaHCO_3_).

^*b*^ Enzymes were pre-incubated with NaHCO_3_ for 10 min to allow optimal carboxylation of lys73, in a similar manner to kinetic determinations, as previously shown (6–8, 20).

## DISCUSSION

The occurrence of OXA-48-R214G (OXA-244) and OXA-181-R214G (OXA-484) is increasingly documented across the world, especially in Europe (2, 14, 16). This is quite surprising as they are loss of function variants, with overall reduced catalytic efficiencies for most β-lactams (6, 8). Possible explanations are difficulties in detection, as these variants do not grow on selective media containing temocillin or ertapenem, thus resulting in silent spread (11, 12). Additionally, it has been suggested that the chromosomal integration of OXA-48-R214G (OXA-244) into the high-risk clone *E. coli* ST38 may also be a driver of the spread, as it stabilizes the genetic traits and reduces the fitness cost (21). While these observations may explain the current spread of these variants, they do not explain their emergence and initial selection.

Here, we showed that R214G variants have increased piperacillin hydrolytic activity, which may have led to their selection using piperacillin, or more likely piperacillin/tazobactam, an antibiotic frequently used in many hospitals all over the world. In France, piperacillin-tazobactam was the fourth most prescribed antibiotic, just behind amoxicillin-clavulanic acid, amoxicillin, and ceftriaxone (17). Piperacillin/tazobactam has already been pointed out as an antibiotic that promotes the selection of expanded-spectrum resistant *E. coli* (19, 22, 23). The increased hydrolysis of piperacillin resulted in higher MICs for the R214G variants against both piperacillin and piperacillin-tazobactam. Nonetheless, no difference was observed for the IC50 of tazobactam. These results suggest that the increased MICs of piperacillin-tazobactam for the R214G variants are solely due to the enhanced catalytic efficiency for piperacillin, which stems from a lower *K_m_*, in agreement with the AI co-folding calculations (Fig. 1).

Superposition of the OXA-244 K73A, OXA-232, and OXA-48 crystal structures reveals a conserved class D β-lactamase scaffold and catalytic core but highlights a marked difference in the β5–β6 loop: in OXA-244, this loop adopts a more open conformation than in OXA-232 and OXA-48 (Fig. 2). Because the β5–β6 loop helps shape the rim of the active-site cleft and influences the substrate entry path, its outward displacement in OXA-244 is expected to enlarge the accessible volume and create a more permissive binding groove. This remodeling is particularly relevant for piperacillin, whose bulky ureidopenicillin side chain typically requires an accommodating, solvent-exposed channel to adopt a productive pre-acylation pose. An open β5–β6 loop in OXA-244 would therefore be expected to reduce steric constraints and improve shape complementarity, enabling additional stabilizing contacts along the side-chain trajectory and favoring formation of the Michaelis complex (Fig. 1). Consistent with this structural interpretation, the more open active-site contour in OXA-244 provides a plausible basis for its higher affinity for piperacillin relative to OXA-232 and OXA-48.

Unlike class A and C serine β-lactamases, the class D β-lactamases have a characteristic hydrolytic mechanism involving a carboxylated lysine residue (K73), which is believed to function as the catalytic base, both during initial acylation of the active site serine and during hydrolysis of the intermediate acyl-enzyme (24–26). In the crystal structure of OXA-48, the side chain of K73 was also carboxylated with carbon dioxide from the medium. This carboxylate group activates the nearby water in the deacylation mechanism (9). The addition of NaHCO_3_ has been shown to increase the hydrolytic activity of OXA-48 β-lactamases and to reduce the biphasic behavior of the hydrolysis for certain substrates (5, 9, 10, 25). Here, we show that the addition of NaHCO_3_ has no impact on the increased affinity but tends to improve the *k_cat_* for piperacillin, with the exception of OXA-48 D159N (OXA-933), for which changes in affinity toward specific substrates are observed. Our results show substrate-dependent variations in OXA-48-like enzymes with respect to the benefit of NaHCO_3_ addition. Specifically, NaHCO_3_ markedly enhanced piperacillin hydrolysis (K*_cat_*/K*_m_*) by OXA-48 (fivefold increase), whereas its effect was considerably weaker on OXA-48 R214G (OXA-244; twofold increase only). This difference might be due directly to the change of the side chain in position 214 and/or to the conformational change of the β5–β6 loop induced by the R214G mutation, as shown in our crystallographic structure of the K73A synthetic mutant of OXA-244 (Fig. 2). The β5–β6 loop may be involved in carbon capture, and hydrolytic activity may vary in the presence of NaHCO_3_, potentially in a substrate-dependent manner.

In addition, we have shown that the R214G AA change in OXA-48 stabilizes the overall protein structure, even though it loosens the active site organization. The increased stability could also contribute to the spread of these types of enzymes. Furthermore, analysis of the MICs in *E. coli* HB4, which is deleted in porins OmpF and OmpC, indicated that the MICs for piperacillin and piperacillin/tazobactam are very high, but as with *E. coli* TOP10, it is the R214G variants that have the highest MICs. OXA-48, as most OXA-type β-lactamases are only weakly inhibited by classical inhibitors such as clavulanic acid or tazobactam (4, 27). In contrast to *E. coli* TOP10, where tazobactam has a moderate impact on piperacillin MICs, the addition of tazobactam does not impact the MICs for piperacillin for *E. coli* HB4. This absence of visible tazobactam inhibitory activity may be due to the already very high MICs, tazobactam activity being negligible, or tazobactam exclusively passing through one of the deleted porins (28, 29). *E. coli* ST38 and ST410, which are responsible for the spread of OXA-48-R214G variants, carry mutations in the OmpC and OmpF porins that could thus reduce membrane permeability toward piperacillin and tazobactam (30). The OmpC ST38-like is a characteristic of ST38 and not widely shared beyond this sequence type, whereas the OmpC R195L mutation is prevalent within ST410 (31).

The increased piperacillin hydrolysis observed for R214G variants may suggest a potential selective advantage at early stages. Nevertheless, the detection of these variants within high-risk clones, together with limitations in clinical detection, indicates that their emergence and dissemination are likely driven by a combination of factors, in which enhanced piperacillin hydrolysis may represent one of several potential contributing elements.

## MATERIALS AND METHODS

### Bacterial strains

*E. coli* TOP10 (Invitrogen, Saint-Aubin, France) and *E. coli* BL21 (DE3; Novagen, VWR International, Fontenay-sous-Bois, France) were used for cloning and overexpression experiments, respectively. *E. coli* HB4 lacking the major porins OmpF and OmpC was also used as a recipient strain to evaluate the relative contribution of these genes when expressed in an *E. coli* isolate with reduced outer membrane permeability (6).

*E. coli* Top10 harboring plasmids pTOPO-OXA-48, pTOPO-OXA-48 R214G (OXA-244), pTOPO-OXA-48 D159N (OXA-933), pTOPO-OXA-181, pTOPO-OXA-181 R214G (OXA-484), and pTOPO-OXA-181 R214S (OXA-232) (6–8, 20), corresponding to pCR-Blunt II-TOPO plasmid (Invitrogen, Illkirch, France) containing the entire coding sequence of *bla*_OXA-48_, *bla*_OXA-48 R214G (OXA-244)_, *bla*_OXA-48 D159N (OXA-933)_, *bla*_OXA-181_, *bla*_OXA-181 R214G (OXA-484)_, and *bla*_OXA-181 R214S (OXA-232)_ genes, respectively, were used for disk diffusion antibiograms (Table 1).

*E. coli* strain BL21 (DE3) harboring plasmids pET41b-OXA-48, pET41b-OXA-48 R214G (OXA-244), pET41b-OXA-48 N159D (OXA-933), pET9a-OXA-181, pET9a-OXA-181 R214G (OXA-484), and pET9a-OXA-181 R214S (OXA-232) containing genes encoding the mature OXA-48, OXA-48 R214G (OXA-244), OXA-48 N159D (OXA-933), OXA-181, OXA-181 R214G (OXA-484), and OXA-181 R214S (OXA-232; from amino acid 20–265) (6–8, 20) were used for β-lactamase purification.

### Susceptibility testing and carbapenemase detection

Antimicrobial susceptibilities were determined by the disk diffusion technique on Mueller-Hinton agar (Bio-Rad, Marnes-La-Coquette, France), and MIC values were determined using the MIC Test Strips (Liofilchem, Roseto degli Abruzzi, Italy), *E*-tests (BioMérieux, Paris, France), and broth microdilution for piperacillin and piperacillin-tazobactam (Sigma–Aldrich, Saint-Quentin-Fallavier, France). Susceptibility results were interpreted according to the EUCAST breakpoints, updated in 2025 (http://www.eucast.org). Detection of a carbapenemase activity was carried out with the Carba NP test as previously described (32). Lateral flow immunoassays were carried out using NG-Test CARBA 5 (NG Biotech, Guipry, France) according to the manufacturer’s instructions (33).

### β-lactamase production and purification

Overnight cultures of *E. coli* BL21(DE3) harboring recombinant plasmid pET41b-OXA-48, pET41b-OXA-48 R214G (OXA-244), pET41b-OXA-48 D159N (OXA-933), pET9a-OXA-181, pET9a-OXA-181 R214G (OXA-484), and pET9a-OXA-181 R214S (OXA-232) were used to inoculate 2 L of lysogeny broth containing 50 mg/L of kanamycin. Bacteria were grown at 37°C until the culture reached an OD_600_ of 0.6. Expression of OXA-variants was induced overnight at 25°C with 0.2 mM isopropyl β-d-1-thiogalactopyranoside (IPTG). OXA-variants were purified as previously described (5). The protein concentrations were determined by measuring the OD at 280 nm and using the extinction coefficients obtained from the ProtParam tool (https://web.expasy.org/protparam/).

### Steady-state kinetic parameters

Steady-state kinetic parameters of purified OXA-48 variants were determined at 30°C in 100 mM sodium phosphate buffer (pH 7.4) supplemented by 25 mM NaHCO_3_ as previously described (6–8, 20). IC50 for inhibitors (MedChemExpress, Monmouth Junction, NJ, USA) were determined in 100 mM sodium phosphate buffer (pH 7) supplemented or not with 25 mM NaHCO_3_ and 100 µM benzylpenicillin as a reporter substrate (Sigma–Aldrich, Saint-Quentin-Fallavier, France).

### Molecular modeling and X-ray crystal structure determination and analysis

Protein-ligand complexes of OXA-48, OXA-48-R214S (OXA-1167), and OXA-48-R214G (OXA-244) with piperacillin were generated using an AI co-folding approach with Chai-1 (34) to identify the structural determinants likely at the origin of the experimentally determined results of piperacillin hydrolysis.

The OXA-244 K73A protein was concentrated to 15 mg/mL in 25 mM HEPES buffer at a pH of 7 (adjusted with KOH) and 50 mM K_2_SO_4_. Crystallization assays were performed using the sitting-drop vapor-diffusion method in which 0.1 µL protein solution and 0.1 µL reservoir solution were equilibrated over 100 µL reservoir solution in 96-well plates at 19°C using a Mosquito HTS robot (SPT Labtech) at the I2BC crystallization facility. The mounted crystal was obtained from condition A4 of the commercial screening kit AmSO4 Suite from Qiagen, consisting of 0.2 M ammonium phosphate and 2.2 M ammonium sulfate. Prior to diffraction analysis, the crystal was cryoprotected by transferring it into a droplet comprising its crystallization solution supplemented with 25% glycerol and was flash cooled in liquid nitrogen. Crystallization information is summarized in Table S1. Diffraction data were collected on the PROXIMA-2 beamline at the SOLEIL synchrotron (Saint-Aubin, France) at a cryogenic temperature of 100 K and were indexed, integrated, and scaled by the automated pipeline using *XDSME* (*XDS Made Easier*; https://github.com/legrandp/xdsme; [35]). Data collection and processing statistics are summarized in Table S2. The solvent content was analyzed using the *CCP*4 program *MATTHEWS_COEF* (36) with a Matthews coefficient of 2.88 for a corresponding ca. 57% solvent. Data collection and processing statistics are summarized in Table S2. Phasing was performed by molecular replacement using *MrBUMP* (37), executing Phaser (38), and using the atomic coordinates of the Class D beta-lactamase PDB entry 6pxx (39) as the best search model. Model refinement was conducted with *BUSTER* (40) using translation–libration–screw motion A and B chains, automated non-crystallographic symmetry restraints, and local structure similarity restraints interspersed with manual adjustments using Coot (41). Two protein residues have alternate conformations, both on the backbone and/or on the Cβ atoms. Structural validation using MolProbity (42) showed 97.14% of the residues in the favored regions of the Ramachandran plot, with a MolProbity score of 1.27 (99th percentile relative to the cohort of PDB structures within 0.25 Å of the file’s resolution; Table S3). The final structure was deposited in the Protein Data Bank (PDB [43]) as entry 9T06. Representations of the structure were generated with UCSF ChimeraX (44) and Pymol (Available at: http://www.pymol.org/pymol.).

### Nano DSF

The thermal denaturation was evaluated using nano DSF using a Tycho NT.6 (NanoTemper Technologies, Munich, Germany) by measuring the intrinsic fluorescence of tryptophan as recommended by the manufacturer.

## SUPPLEMENTAL MATERIAL

Supplemental material aac.01267-25-s0001.docx Tables S1–S3.

ASM does not own the copyrights to Supplemental Material that may be linked to, or accessed through, an article. The authors have granted ASM a non-exclusive, world-wide license to publish the Supplemental Material files. Please contact the corresponding author directly for reuse.
