# Supplementary material for: Improved hydrolysis of piperacillin by OXA-48-like R214G variants, a selective advantage under piperacillin-tazobactam exposure
Source: Antimicrob Agents Chemother. 2026 Apr 29;70(6):e01267-25. doi: 10.1128/aac.01267-25 (PMC13231874; doi:10.1128/aac.01267-25)
Supplement: Supplemental material — Tables S1–S3. [file aac.01267-25-s0001.docx]

**Revised AAC01267-25**

**Supplementary information**

**Improved hydrolysis of piperacillin by OXA-48-like R214G variants, a selective advantage under piperacillin-tazobactam exposure**

Réva NERMONT,^a^ Saoussen OUESLATI,^a,b^ Magali AUMONT-NICAISE,^c^ Pascal RETAILLEAU,^d^ Bogdan I. IORGA,^d^ Thierry NAAS^a,b,e,*^

*^a^Team 'Resist', UMR1184 'Immunology of Viral, Auto-Immune, Hematological and Bacterial Diseases (IMVA-HB),' INSERM, Université Paris-Saclay, CEA, LabEx LERMIT, Faculty of Medicine, Le Kremlin-Bicêtre, France.*

*^b^Bacteriology-hygiene department, Hôpital Bicêtre, AP-HP Paris-Saclay, 94270 Le Kremlin-Bicêtre, France*

*^c^Université Paris-Saclay, CNRS,* ***Plateforme Interactions des Macromolécules***
***Institut de Biologie Intégrative de la cellule (I2BC) -*** ***UMR9198****, 91190 Gif-sur-Yvette, France*

*^d^Université Paris-Saclay, CNRS, Institut de Chimie des Substances Naturelles (ICSN), 91190 Gif-sur-Yvette, France*

*^e^French National Reference Center for Antibiotic Resistance: Carbapenemase-Producing Enterobacterales, Hôpital Bicêtre, AP-HP Paris-Saclay, 94270 Le Kremlin-Bicêtre, France*

* Corresponding author:

- Thierry Naas: Phone: 33-1-45-21-29-86. Fax: 33-1-45-21-63-40. E-mail: [thierry.naas@aphp.fr](mailto:thierry.naas@aphp.fr)

| **Table S1** Crystallization conditions |
| --- |

|  |
| --- |

| Method | Vapour diffusion |
| --- | --- |
| Plate type | Sitting drop 96-well Intelli-Plate (Hampton Research) |
| Temperature (K) | 292 |
| Protein concentration (mg ml^−1^) | 15 |
| Buffer composition of protein solution | 250 m*M* Hepes pH 7.0, 50mM K_2_SO_4_ |
| Composition of reservoir solution | 0.2 *M* ammonium phosphate, 2.2 *M* ammonium sulfate |
| Volume and ratio of drop | 0.2 µl, 1:1 |
| Volume of reservoir (µl) | 100 |

| **Table S2** Data collection and processing |
| --- |

| Values in parentheses are for the outer shell. |
| --- |

| Diffraction source | PROXIMA-2A, SOLEIL |  |
| --- | --- | --- |
| Wavelength (Å) | 0.95372 |  |
| Temperature (K) | 100 |  |
| Detector | EIGER X 9M |  |
| Crystal-to-detector distance (mm) | 200.0 |  |
| Rotation range per image (°) | 0.1 |  |
| Total rotation range (°) | 360 |  |
| Space group | *P4*_3_2_1_2 |  |
| *a*, *b*, *c* (Å) | 90.54 90.54 163.47 |  |
| α, β, γ (°) | 90, 90, 90 |  |
| Mosaicity (°) | 0.05 |  |
| Isotropic processing | |  |
| Resolution range (Å) | 46.67 - 1.84 (1.96 - 1.84) |  |
| Total No. of reflections | 1596596 (4033) |  |
| No. of unique reflections | 59031 (842) |  |
| Completeness (%) | 99.9 (99.4) |  |
| Multiplicity | 27 (4.8) |  |
| 〈*I*/σ(*I*)〉 | 29.38 (3.13) |  |
| *R*_r.i.m._ | 0.079 (0.905) |  |
| *R_sym_* | 0.077 (0.889) |  |
| Overall *B* factor from Wilson plot (Å^2^) | 37.3 |  |
| CC_1/2_ | 1.000 (0.964) |  |

**Table S3**
Refinement

| Rcryst/Rfree | 0.199/0.219 |
| --- | --- |
| No. of reflections for Rcryst/Rfree | 59001/2973 |
| No. of protein residues | 494 |
| No. of SO_4_^2−^ ions / glycerol solvent molecules | 14 (8 with half-occ.) / 3 |
| No. of water molecules | 477 |
| Cruickshank DPI (Å) | 0.123 |
| R.m.s.d., bond lengths (Å) | 0.008 |
| R.m.s.d., angles (°) | 0.92 |
| R.m.s.d., peptide omega torsion angles (°) | 3.44 |
| Average *B* factors (Å^2^) | |
| Overall | 44.16 |
| Water molecules | 54.04 |
| Solvent molecules | 65.3/67.8 |
| Ramachandran analysis | |
| Residues in favoured regions (%) | 97.1 |
| Cis-Prolines (%) | 2/12 |
| Poor Rotamers (%) | 1.17 |
